# Relatives’ experiences of visiting restrictions during the COVID-19 pandemic’s first wave: a PREMs study in Valais Hospital, Switzerland

**DOI:** 10.1186/s12913-023-10013-9

**Published:** 2023-09-19

**Authors:** N. Tacchini-Jacquier, S. Monnay, E. Bonvin, J. Dubuis, H. Verloo

**Affiliations:** 1 Development of Nursing Practices Unit, Valais Hospital, 86, Avenue du Grand-Champsec, CH–1951 Sion, Switzerland; 2 Social Affairs and Human Resources Specialist, Valais Hospital, 86, Avenue du Grand-Champsec, CH–1951 Sion, Switzerland; 3 Valais Hospital, 86, Avenue Grand-Champsec, CH–1951 Sion, Switzerland; 4 Valais Family Caregivers’ Association, 19, Avenue de Tourbillon, CH-1950 Sion, Switzerland; 5 Valais Hospital, HES-SO Valais/Wallis, 5, Chemin de L’Agasse, CH–1950 Sion, Valais Switzerland

**Keywords:** Visitors to patients, COVID-19, SARS-CoV-2, Pandemic, Health policy, Caregivers, Family, Public health

## Abstract

**Background:**

During the COVID-19 pandemic, most countries introduced temporary visiting restrictions on the relatives of acute care hospital patients, whether or not they were infected with SARS-CoV-2. This affected relatives’ psychological and emotional states and how closely they could be involved in their loved one’s hospitalization.

**Study aims:**

Investigate relatives’ experiences of visiting restrictions during the COVID-19 pandemic’s first wave and the support offered by Valais Hospital’s healthcare staff.

**Methods:**

Relatives and patients who had been discharged between February 28 and May 13, 2020, were asked to complete a patient-reported experience measures (PREMs) questionnaire, whether or not they had been infected by SARS-CoV-2. Relatives were asked about how visiting restrictions had affected them, their perceptions of the severity of the COVID-19 pandemic, the quality of communication concerning their loved ones’ health status during their hospitalization, and the information received from healthcare staff. Descriptive and inferential statistics were computed.

**Results:**

Of 866 PREMs questionnaires returned, 818 were analyzable, and 543 relatives had experienced visiting restrictions to their loved ones: 92 relatives (87%) of COVID-19 patients and 451 relatives (66%) of non-infected patients, with heterogenous effects on their psychological and affective status. Overall, whether or not relatives were subjected to visiting restrictions, they perceived themselves to be well treated, well informed, and that communication with hospital healthcare staff was satisfactory. However, relatives subjected to visiting restrictions reported significantly lower scores on the quality of communication than other relatives. The relatives of patients in gynecology/obstetrics and internal medicine wards were significantly more affected by visiting restrictions than were the relatives of patients in other wards. Numerous relatives subjected to visiting restrictions reported regular communication with their loved ones or with healthcare staff, at least once a day (*n* = 179), either via videoconferences using FaceTime®, WhatsApp®, Zoom®, or Skype® or via mobile phone text messages.

**Conclusion:**

Visiting restrictions affected relatives differently depending on the wards their loved ones were hospitalized. Healthcare institutions should investigate the utility of visiting restrictions on patients, how they affect relatives, and how to improve personalized patient–relative communications. Future research should attempt to develop reliable, validated measurement instruments of relatives’ experiences of acute-care visiting restrictions during pandemics.

**Supplementary Information:**

The online version contains supplementary material available at 10.1186/s12913-023-10013-9.

## Introduction – background

The first cases of COVID-19 struck the Canton of Valais, Switzerland, at the end of February 2020. The Swiss Confederation and the Canton of Valais enacted significant measures to limit the virus’ spread throughout the population, including a reorganization of acute hospital care [1]. During the onset of the pandemic, from March 15 to April 30, 2020, the Canton instituted COVID-19-related visiting restrictions on healthcare institutions, prohibited patients from leaving their rooms, and closed hospital restaurants, coffee shops, and other communal areas. All visits were banned, including by relatives, with some exceptions made for parents visiting pediatrics wards [2]. These decisions were made when knowledge about the virus’ spread was not very advanced regarding patient safety [3]. Restricting hospital visits during the COVID-19 pandemic’s first wave had the following aims: (1) preventing the transmission of SARS-CoV-2 from the community into acute hospitals (infecting healthcare staff and patients), (2) preventing transmission in the other direction (infecting visitors), and (3) maintaining adequate supplies of personal protective equipment [4]. At the pandemic’s onset, potential visitor-related COVID-19 outbreaks were considered a substantial risk and, thus, all types of visits were restricted, despite a lack of scientific evidence linking visitors to SARS-CoV-2 transmission in hospitals [5, 6].

Restricting visits is not only an emotional hardship for patients and relatives, but healthcare staff also perceive the absence of relatives at the bedside to be a hindrance to delivering person- and family-centered care [7, 8]. The present study defined relatives as the non-professional persons providing physical help and psychological support to patients, and they could be family members, friends, or acquaintances [9]. Patient accompaniment by healthcare staff can be conceptualized as social, emotional (e.g., moral support), and informational support (e.g., helping to facilitate patient–healthcare staff communication) that increases beneficial health outcomes. Accompanying patients in those three dimensions may not always be feasible for many relatives due to work or other responsibilities during hospitalization. However, accompaniment by relatives can significantly influence chronic illness self-care. The presence of relatives facilitates communication between patients and healthcare professionals and enhances patients’ satisfaction with them. Understanding how the mechanisms of relatives’ involvement influences care and outcomes is critical to better understanding the concept of visiting restrictions [10]. Under normal circumstances, relatives at the bedside can observe how different healthcare staff care for their loved ones [11]. Depending on the patient’s disabilities and unique needs, relatives can learn how to assist their loved ones in the activities of daily living and note whether they are experiencing discomfort. Learning how to react at the bedside enables relatives to become accustomed to the patient’s changing condition and better help manage discharge planning and support needs [12]. Research has shown the significant numbers of medical and nursing tasks performed by relatives at home with limited guidance [13]. However, some care situations could have been exacerbated by COVID-19 visiting restrictions [14]. A relative can help overcome language barriers and health literacy problems caused by clinical jargon [15] or can assist physically weakened and/or mentally inhibited patients [16, 17]. Previous studies have also demonstrated that relatives are crucial to the early detection of delirium, a common, often unrecognized condition present in frail older adult inpatients diagnosed with dementia or multiple other chronic conditions and polypharmacy [18, 19]. The regular presence of relatives at the bedsides of those patients most at risk of delirium can reduce its onset and limit long-term functional decline [15, 19]. Recent data revealed that the longer the hospital length of stay (LOS), the more relatives were emotionally affected by visiting restrictions [20, 21]. Relatives also take on an advocacy role when they communicate practical suggestions about patients’ habits or additional needs to healthcare staff, thus facilitating patient–staff communication [22]. Sahoo et al. and Vincent et al. (2021) reported significant associations between additional stress, affect, visiting restrictions, and LOS [23–25]. The COVID-19 pandemic also changed patient discharge planning, undermining usual discharge processes. Before the COVID-19 pandemic, healthcare staff used a discharge procedure designed to bring relatives and the patient together to discuss critical information on the support that would be needed at home [26]. This exchange increased the chances of the patient subsequently remaining at home and optimized the discharge process. Under COVID-19 visiting restrictions, these conversations altered dramatically [27] and may have caused problems in the dialogue between healthcare staff and relatives, reducing the possibilities of ensuring consensus-based care and increasing the risks of unplanned hospital readmissions [26, 28]. To maintain the links between patients, relatives, and healthcare staff, the Valais Hospital offered a variety of digital and technical means to replace physical visits [29, 30]. However, recent studies have highlighted that video or telephone meetings with the relatives of patients in acute care settings led to fewer changes to care goals than in-person meetings [31–34]. Substitute visiting methods, such as digital and multimedia applications, lowered relatives’ comprehension of the patient’s overall condition, reducing opportunities to maintain social relations [25, 26]. Recent research has shown that relatives’ experiences during the uncertain context of COVID-19 led to frustrations, especially among older adults [35, 36]. This was linked to not being able to see how their loved one was being cared for and having to put their trust in healthcare institutions [37]. Unclear information or inconsistencies in institutional policy contributed to these uncertainties and relatives sought efficient face-to-face communication [37]. Hoffman et al. highlighted the need for personal attention from relatives [37–39]. To assess inpatients’ relatives’ experiences with regard to the visiting restrictions imposed during COVID-19’s first wave, we distributed a patient-reported experience measures (PREMs) questionnaire to all the patients hospitalized in the Valais Hospital between the end of February and mid-May, 2020, and to their relatives. The following research questions guided this research. How were the relatives subjected to visiting restrictions distributed? How were the relatives subjected to visiting restrictions affected by this situation compared to relatives not subjected to visiting restrictions? How did relatives (whether subjected to visiting restrictions or not) perceive the information they received, communication with staff, and their own involvement in the care of their loved ones? How did relatives maintain contact with their loved ones?

## Methods

### Design, research population, and setting

Following the approval from the Human Research Ethics Committee of the Canton of Vaud (2020–02025), Valais Hospital’s data science warehouse provided the contact details of all the adult inpatients (18 years and older) discharged alive to their home or a nursing home between February 28 and May 13, 2020. These were extracted from administrative, electronic patient records in the hospital’s patient register. A paper questionnaire was sent out to these patients, including an explanation sheet describing the nature of the survey and a questionnaire for their relative, if appropriate. Patients were free to choose whether to participate. Anonymously returning the questionnaire in the attached postage-paid envelope was considered consent to participate in our study for both patients and relatives. The previously published research protocol describes the PREMs methodology used for our survey [40].

### Study framework

**Fig. 1:**
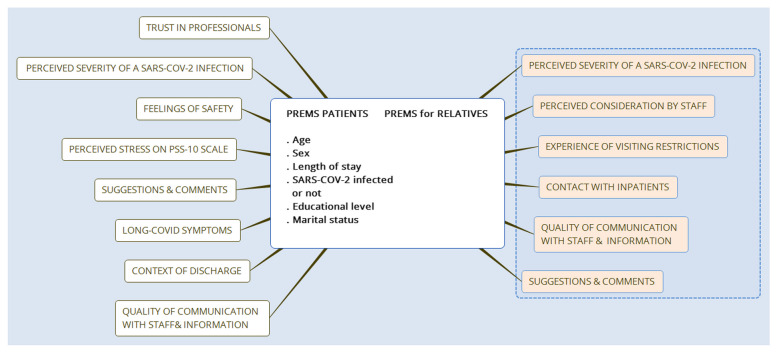
The study’s patient-reported experience measures (PREMs) framework and the reported data inside the blue frame

The Quadruple Aim healthcare framework guided the study, highlighting the medical and social needs of hospitalized patients and their relatives, emphasizing the impacts of their unmet needs, and describing the importance of partnerships between the healthcare system and formal and informal caregivers [29]. Relatives involved in care delivery have also recently become an acknowledged essential component of overall health system performance, based on the principles of patient and public involvement described in PREMs [30, 31]. PREMs instruments look at the care process’s impact on patients’ and relatives’ experiences, e.g., involvement in care, communication with staff, information sharing, and the overall care experience. Our PREMs questionnaire included open and closed questions to capture patients’ and relatives’ perceptions of their interactions with the healthcare system and the degree to which their needs were considered [41]. This paper reports on the PREMs survey’s written feedback on relatives’ experiences during the COVID-19 pandemic’s first wave and the visiting restrictions imposed on them (Fig. 1).

### The PREMs instrument

Our self-reporting data-collection questionnaire was designed based on a literature review and four semi-structured exploratory interviews with previously hospitalized patients and their relatives [20, 42, 43]. A returned questionnaire from the patient and relative served as a proxy for written consent to participate. The first section, including 14 closed questions, asked patients about sociodemographic data, sex, age, marital status, educational level, and their hospital trajectory as a patient, as well as about their stress level [44], trust in healthcare professionals (nurses and physicians) [45], feelings of safety [46], whether they had been infected by SARS-CoV-2, and perceptions about the disease’s severity during the hospitalization period [47]. The second section included eight closed questions and one open-ended question for the discharged patient’s relative (if they were directly involved in the patient’s hospitalization) (Additional file 1). Due to legal restrictions covering data protection and confidentiality, we were not allowed to collect sociodemographic data on relatives. These questions were: Were you able to visit your relative in the hospital? [Yes/No]; If not, how did you maintain contact with your loved one? [(i) Telephone with professional caregivers, (ii) email, (iii) other]. If not, how much did this affect you? [(i) I was not affected, (ii) I was slightly affected, (iii) No opinion, (iv) I was moderately affected, (v) I was very affected]; How did you perceive the information you received about the COVID-19 pandemic during your loved one’s hospital stay? [(i) Totally inadequate, ii) Inadequate, iii) Slightly inadequate, iv) No opinion, v) Just good enough, vi) Adequate, vii) Very good)]; How would you rate communication with the staff? [(i) Poor, ii) Passable, (iii) Good, (iv) Very good, (v) Excellent]. As a close relative, how did the hospital staff treat you? [(i) I was not taken into consideration at all, (ii) I was moderately taken into consideration, (iii) I was fully taken into consideration]. How serious do you think the COVID-19 pandemic is? [(i) Not at all serious, (ii) Not very serious, (iii) Slightly serious, (iv) Serious, (v) Very serious] [47]. Would you like to add any comments about your experience of your loved one’s hospitalization during the pandemic?

### Data collection procedure

**Fig. 2:**
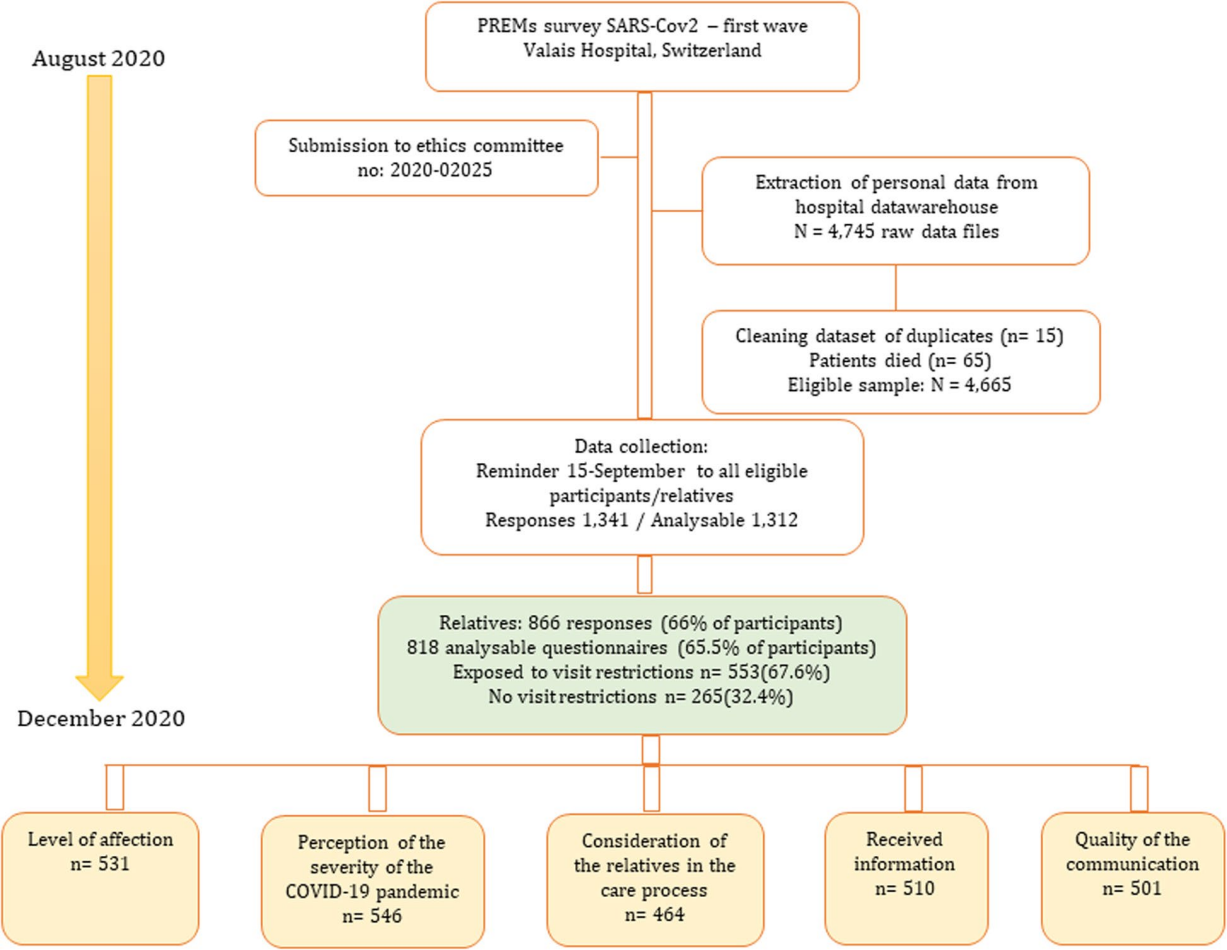
Strategy for data collection from patients and relatives

All eligible participants received a letter by post inviting them to participate in the survey. This was followed by a reminder two weeks later. Besides the paper questionnaire, an information sheet explained the study’s background, the data sought, and our participant data protection strategy. Participants were asked to complete the paper questionnaire and return it in the prepaid envelope provided. Waiting for ethics clearance and heavy workloads meant that the data warehouse only started its information gathering in August and finished in December 2020 (Fig. 2).

### Data analyses

Data were anonymized to ensure participant anonymity and respect good research practice in this type of study, as per the Declaration of Helsinki [35]. Data were imported into IBM SPSS® software, version 28 (IBM Corp, Armonk, New York, USA), for analyses. Our statistical power calculation was based on an alpha error of 0.01, a power-type II error of 0.99, and a mild effect size of 0.3. The minimum sample required for sufficient statistical power was 740 relatives. We analyzed the number of responses and missing values for each variable and reported them in our tables (*n* = answers). Parametric properties were analyzed for the normality of their distributions and the equality of their variances using the Kolmogorov–Smirnov test. Non-parametric tests were performed for variables with non-normal distributions to compare relatives who were and were not affected by visiting restrictions. The population was described using descriptive statistics with frequencies, distributions, and leading trends. Data collected using Likert scales were analyzed using descriptive and inferential statistics. LOS was recoded as a dichotomous variable of 1–14 days and ≥ 15 days, based on the median patient LOS [23, 25]. Bivariate analyses were conducted using cross-tabulations between relatives impacted and not impacted by visiting restrictions during their loved one’s hospitalization. Spearman’s rank correlation measures were computed between sociodemographic variables and the closed questions. We computed a linear multivariate regression model to analyze how visiting restrictions predicted relatives’ affects, their satisfaction with information received about the COVID-19 pandemic, satisfaction with communication with staff, how well healthcare staff considered relatives, and perceptions of how serious the COVID-19 pandemic was. The model estimated each predictor’s net impact, other things being equal, and it gave predictions for the entire sample, not just specific individuals. A content analysis [48] of relatives’ responses to the open-ended question was made using NVivo12 software (QSR International, 2021).

Quantitative results were considered statistically significant when *p* < 0.01. All *p*-values were based on two-tailed tests, and all the analyses were supervised and reviewed by a biostatistician.

## Results

### Study participants

Of 4,523 eligible participants hospitalized during the COVID-19 pandemic’s first wave, 1,341 (29.6%) returned the questionnaire. Of these, 1,312 were valid (> 50% of questions completed), with 866 relatives completing the section dedicated to them, 818 of which were analyzable (> 50% of questions completed), representing 65.5% of the valid patient responses.

### Sociodemographic characteristics of patients and their relatives

#### Participants – hospitalized patients

Median participant age was 64 years old (IQR 1–3 = 45–76). During the study period, 141 (10.9%) respondents were tested positive for a SARS-CoV-2 infection by the hospital laboratory, and 1,148 (89.1%) were uninfected. Discharged patients’ sociodemographic data are detailed in Table 1.

**Table 1:** Participating patients’ sociodemographic and hospitalization characteristics

**Characteristics**	**Participants**
**Age (years) (*n* = 1,195)**	
Mean (SD)	60.3 (19.4)
Median (IQR 1–3)	64 (45–76)
Min–Max	18–99
**Age groups (years) (*n* = 1,195)**	
18–34 (%)	185 (15.5)
35–55 (%)	238 (19.9)
56–64 (%)	183 (15.3)
65–74 (%)	253 (21.2)
75 or more (%)	336 (28.1)
**Sex (*n* = 1,291)**	
Male (%) / Female (%)	619 (47.9) / 672 (52.1)
**Marital status (*n* = 1,161)**	
Single (%)	451 (34.3)
Married (%)	579 (44.1)
Divorced/separated (%)	131 (10.0)
Widowed (%)	84 (6.4)
**Educational level (*n* = 1,211)**	
Compulsory education (%)	375 (28.6)
Secondary education (%)	547 (41.7)
Higher education/university (%)	289 (22.0)
**Length of stay (days) (*n* = 1,080)**	
Mean (SD)	15.1 (26.7)
Min–Max	1–280
**SARS-CoV-2-infected (*n* = 1,289)**	
Yes (%) / No (%)	141 (10.9) / 1,148 (89.1)
**Hospitalization ward (*n* = 1,312)**	
Surgery (%)	300 (22.9)
General Medicine (%)	240 (18.3)
Gynecology/obstetrics (%)	169 (12.9)
Intermediate care & ICUs (%)	230 (17.5)
Psychiatry (%)	44 (3.4)
Rehabilitation/geriatrics (%)	24 (1.8)
Other units (%)	228 (17.4)
Complex trajectory^a^ (%)	77 (5.9)

^a^Two hospital units or more

#### Responding relatives

Of 866 PREMs questionnaires completed by patients’ relatives, 818 (95%) were analyzable, including 106 (75%) relatives of the 141 SARS-CoV-2-infected participants. Among the 1,086 non-COVID-19 participants, 712 (87%) relatives responded to the PREMs questionnaire’s second section. We found significantly higher survey participation rates among the relatives of: patients infected with SARS-CoV-2 (*p* < 0.001); older patient age groups (*p* = 0.008); patients with longer LOS (*p* < 0.001); and patients in certain hospital wards (intermediate care and ICU) (*p* < 0.001).

##### Visiting restrictions

A total of 543 relatives were subjected—either entirely or partially—to visiting restrictions during their loved one’s hospitalization, including 92 (87%) relatives of SARS-CoV-2-infected patients and 451 (63%) relatives of non-infected patients. Relatives of SARS-CoV-2-infected patients were significantly more emotionally affected than the relatives of non-infected patients (81% vs. 61% at least moderately affected, respectively; *p* < 0.001) (Table 2). Contrarily, no significant differences were found between how strongly relatives subjected to visiting restrictions were affected according to age group (*p* = 0.815) and LOS (*p* = 0.185) (Table 2).

**Table 2:** Distribution of loved ones’ levels of affection due to visiting restrictions

**Variables**	**No opinion n (%)**	**Not affected n (%)**	**Mildly affected n (%)**	**Moderately affected n (%)**	**Strongly Affected n (%)**	**Median (IQR 1–3)**	***p*-value**
**Visiting restrictions (*n* = 531)**	51 (9.6)	83 (15.6)	54 (10.2)	96 (18.1)	247 (46.5)	4 (2–5)	< 0.001^*^
SARS-CoV-2-infected patients (*n* = 91)	7 (7.7)	5 (5.5)	5 (5.5)	14 (15.4)	60 (65.9)	5 (4–5)	
Non-infected patients (*n* = 430)	44 (10.2)	75 (17.4)	48 (11.2)	79 (18.4)	184 (42.8)	4 (2–5)	
**Inpatient age group (years) (*n* = 487)**							0.815^**^
18–34 (*n* = 71)	6 (8.5)	11 (15.5)	6 (8.5)	9 (12.7)	39 (54.9)	5 (3–5)	
35–55 (*n* = 92)	8 (8.7)	12 (13)	9 (9.8)	18 (19.6)	45 (48.9)	4 (3–5)	
56–64 (*n* = 72)	6 (8.3)	11 (15.3)	9 (12.5)	16 (22.2)	30 (41.7)	4 (3–5)	
65–74 (*n* = 107)	11 (10.3)	13 (12.1)	14 (13.1)	18 (16.8)	51 (47.7)	4 (3–5)	
75 or more (*n* = 145)	10 (6.9)	30 (20.7)	10 (6.9)	27 (18.6)	68 (46.9)	4 (2–5)	
**Hospitalization ward (*n* = 531)**							< 0.001^**^
Surgery (*n* = 88)	9 (10.2)	21 (23.9)	14 (15.9)	22 (25.0)	22 (25.0)	4 (2–5)	
General Medicine (*n* = 119)	14 (11.8)	12 (10.1)	13 (10.9)	22 (18.5)	58 (48.7)	4 (3–5)	
Gynecology/obstetrics. (*n* = 64)	3 (4.7)	9 (14.1)	2 (3.1)	8 (12.5)	42 (65.6)	5 (4–5)	
Intermediate care & ICU (*n* = 115)	5 (4.3)	10 (8.7)	15 (13)	23 (20)	62 (53.9)	5 (3–5)	
Unknown trajectory (*n* = 87)	16 (18.4)	23 (26.4)	5 (5.7)	8 (9.2)	35 (40.2)	3 (2–5)	
Psychiatry (*n* = 14)	2 (14.3)	2 (4.3)	0	4 (28.6)	6 (42.9)	4 (2–5)	
Rehabilitation/geriatrics (*n* = 9)	0	0	0	3 (33.3)	6 (66.7)	4 (4–5)	
Multiple wards other than ICU (*n* = 35)	2 (5.7)	6 (17.1)	5 (14.3)	6 (17.1)	16 (45.7)	4 (3–5)	
**Length of stay (days) (*n* = 445)**							0.185^*^
1–14 (*n* = 321)	29 (9)	53 (16.5)	37 (11.5)	37 (11.5)	143 (44.5)	4 (2–5)	
≥ 15 (*n* = 124)	9 (7.3)	14 (11.3)	9 (7.3)	22 (17.7)	70 (56.5)	5 (3–5)	

^*^Non-parametric Mann–Whitney U test

^**^Kruskal–Wallis test

### Relatives’ perceptions of the severity of the COVID-19 pandemic

Using the standard questionnaire and scale for risk perception during an infectious disease outbreak, as developed by the Municipal Public Health Service of Rotterdam-Rijnmond [47], relatives’ overall median score for the perceived severity of a SARS-CoV-2 infection was 4 (IQR 1–3 = 3–5). No significant differences were found between relatives subjected to visiting restrictions (median 4; IQR 1–3 = 3–5) and those not (median 4; IQR = 1–3 = 3–5) (*p* = 0.085).

### Relatives’ involvement in care

#### Consideration of relatives in the care process

Overall, most relatives felt well-considered by healthcare staff (*n* = 406; 54.4%) when it came to involvement in the provision of care. Given the exceptional public health situation caused by the COVID-19 pandemic, relatives waiting to hear from their loved ones felt stressed and disturbed. A smaller fraction felt less well considered (*n* = 218; 29.1%) and 124 (16.6%) did not feel considered at all in the provision of care. A small fraction (< 5%) reported hospital healthcare staff to be unavailable to inform them of their loved one’s health status. Significant differences were found between patient age groups (*p* < 0.001) and between relatives subjected and not subjected to visiting restrictions (*p* < 0.001). No significant differences were found regarding LOS (*p* = 0.060) or hospitalization ward (*p* = 0.316) (Table 3).

**Table 3:** Relatives’ perceptions of being considered as care partners

**Relatives**	**Not considered n (%)**	**Partially considered n (%)**	**Fully considered n (%)**	***P*-value**
**Overall (*n* = 748)**	124 (16.6)	218 (29.1)	406 (54.4)	< 0.001^*^
Subjected to visiting restrictions (*n* = 464)	109 (23.5)	138 (29.7)	217 (46.8)	
Not subjected to visiting restriction (*n* = 284)	15 (5.3)	80 (28.2)	189 (66.5)	
**SARS-CoV-2 (*n* = 736)**				0.350^*^
SARS-CoV-2-infected patients (*n* = 98)	20 (20.4)	23 (23.5)	55 (56.1)	
Non-infected patients (*n* = 638)	103 (16.1)	189 (29.6)	346 (54.2)	
**Inpatient age group (years) (*n* = 696)**				< 0.001^*^
18–34 (*n* = 124)	23 (18.5)	50 (40.3)	51 (41.1)	
35–55 (*n* = 145)	29 (20)	37 (25.5)	79 (54.5)	
56–64 (*n* = 93)	18 (19.4)	33(35.5)	42 (45.2)	
65–74 (*n* = 139)	28 (20.1)	34 (24.5)	77 (55.4)	
75 or more (*n* = 195)	19 (9.7)	52 (25.7)	124 (63.6)	
**Length of stay (days) (*n* = 619)**				0.060^*^
1–14 (*n* = 458)	89 (19.4)	134 (29.3)	235 (51.3)	
≥ 15 (*n* = 161)	21 (13)	41 (25.5)	99 (61.5)	
**Hospitalization ward (*n* = 748)**				0.316^*^
Surgery (*n* = 126)	28 (22.2)	39 (31)	59(46.8)	
General Medicine (*n* = 145)	30(20.7)	40 (27.6)	75 (51.7)	
Gynecology/obstetrics (*n* = 128)	14 (10.9)	46 (35.9)	68 (53.1)	
Intermediate care/ICU (*n* = 147)	21 (14.3)	41 (27.9)	85 (57.8)	
Unknown trajectory (*n* = 115)	18 (15.7)	30 (26.1)	67 (58.3)	
Psychiatry (*n* = 23)	2 (8.7)	8 (34.8)	13(56.5)	
Rehabilitation/geriatrics (*n* = 16)	4 (25)	4 (25)	8 (50)	
Multiple wards other than ICU (*n* = 48)	7 (14.6)	10 (20.8)	31 (64.6)	

^*^Chi-squared exact test

#### Sharing information and communication between healthcare staff and relatives

Despite healthcare staff’s poor availability due to extremely high workloads, most relatives felt well informed by them (*n* = 426; 53.0%), with an overall median score of 6 (IQR 1–3 = 1–6). Fewer respondents felt moderately well informed (*n* = 68; 8.5%) or poorly informed (*n* = 309; 38.5%) by healthcare staff. Among relatives subjected to visiting restrictions, no significant differences were found regarding perceived levels of information between the sexes (*p* = 0.080), between SARS-CoV-2-infected or non-infected patients (*p* = 0.254), between age groups (*p* = 0.248), and between different LOS (*p* = 0.220). Contrarily, significant differences were found between hospitalization wards (*p* < 0.001) (Additional file 2).

Relatives reported a reasonable overall median score of 3 out of 5 (IQR 1–3 = 3–4) on the quality of their communication with hospital healthcare staff, although relatives subjected to visiting restrictions reported significantly lower scores than those not subjected to them (*p* < 0.001). One-fifth of relatives found communication poor or acceptable. No significant differences were found between relatives subjected to visiting restrictions and those not with regards to communication, LOS, and hospitalization wards (Additional file 3). Among the full sample of relatives (*n* = 818), 563 (69%) reported regularly communicating with their hospitalized loved ones (at least once a day), and 179 (22%) reported having at least one telephone contact with Valais Hospital staff. A small number of relatives (*n* = 6) communicated with the patient by email. Other methods for maintaining contact between relatives and patients were videoconferences using FaceTime®, WhatsApp®, Zoom®, or Skype® (*n* = 25), mobile phone and SMS text messages (*n* = 9), exchanges at the hospital window or outside the ward (*n* = 9), being hospitalized in the same hospital room (*n* = 1), or communication through the family physician (*n* = 3).

#### Multivariate linear regressions of affect scores

Simultaneous multiple linear regressions were calculated to investigate the best predictors of affect scores among relatives subjected to visiting restrictions. The combination of patient age in years, sex, LOS, and the hospitalization wards of medicine, surgery, psychiatry, gynecology, intermediate care/ICU, and rehabilitation/geriatrics significantly predicted affect scores (*F *(9, 4.421) = 7.294; *p* < 0.001). The hospitalization wards of medicine (*p* = 0.027) and gynecology/obstetrics (*p* = 0.028) also significantly predicted relatives’ affect scores (Table 4). The adjusted R^2^ value was 0.105, indicating that the model explained 10.5% of the variance in the affect scores. According to Cohen, this is a mild-to-moderate effect [49].

**Table 4:** Multivariate linear regression prediction analyses of relatives’ affect scores

**Variables**	**B**	**Std. Error**	**Exp(B)**	**t**	**Sig**	95% Confidence Interval Beta	
						Under limit	Upper limit
Patient age in years	-.0003	0.004	-0.047	-0.767	0.444	-0.011	0.005
Sex	0.006	0.146	0.002	0.039	0.969	-0.282	0.293
Surgery dept	0.216	0.160	0.074	1.355	0.176	-0.098	0.530
General medicine dept	-0.348	0.157	-0.124	-2.215	0.027^*^	-0.656	-0.039
Psychiatry dept	-.0010	0.399	-0.001	-0.024	0.981	-0.795	0.776
Gynecology/obstetrics dept	-0.528	0.239	-0.145	-2.207	0.028^*^	-0.999	-0.058
Intermediate care/ICU	-0.132	0.195	-0.036	-0.675	0.500	-0.516	0.252
Rehabilitation/geriatrics dept	-0.536	0.243	-0.123	-2.202	0.028	-1.014	-0.057
Length of stay	0.005	0.003	0.091	1.650	0.100	-0.001	-0.011
Intercept	7.607	1.305		5.831	< 0.001	5.042	10.172

R^2^ = 0.105; *F *(9, 4.421) = 7.294; *p* < 0.001

*dpt* department

* = signicantly predict the affect scores of the relatives

#### Relatives’ freely expressed experiences of visiting restrictions

Almost one-fifth (*n* = 71) of the relatives subjected to visiting restrictions described their lived experiences in our open-ended question.

#### Relatives of patients hospitalized in gynecology/obstetrics

Fathers were initially excluded from attending the mother’s initial labor, causing a lot of frustration and stress for both. Relatives understood the need for preventive measures against the SARS-CoV-2 virus, but they did not consider their loved ones as sick patients, finding the prohibition on visiting too extreme. Limitations and even prohibitions on visits by fathers were not well received, especially the time limit of 30 min. Being deprived of this unique life experience, unable to provide support to the mother or see the child’s birth and their first days of life, was a very bad experience for fathers, filled with intense regrets. The following comment summed up the disagreements with maternity ward visiting restrictions: “In the case of childbirth, the father’s place—who could have been tested before—is next to the mother and the child. Don’t you think?” (Relative-223)

#### Neonatology

Limitations and even prohibitions on visiting the neonatology ward were very badly received by relatives. Relatives prohibited from visiting the neonatology unit stated the following:

“Understanding the hospital sector’s state of stress… I had expected a different appreciation of priorities... For me, hospitalization in neonatology should ensure the right to visits no matter what.” (Relative-345)

#### Emergency department visits and the hospitalization of frail subjects

The prohibition on visits also affected relatives accompanying their loved ones to urgent admissions to the emergency department. The moment of this imposed separation—leaving their loved one to the unknown—aroused very strong emotions, including worry, anxiety, stress, the fear of not seeing them again, and intense apprehension while waiting for news. They expressed these emotions as follows:

“The ban on visits is traumatic for all relatives.” (Relative-87)

“It is tough to leave a loved one—especially my sick wife—outside the door without accompanying her or supporting her during these difficult moments, but I understand the measures taken.” (Relative-340)

Visiting restrictions were very badly received by relatives and frail patients alike, especially when involving patients with cognitive disorders or at the end of life, with whom video calls were complicated or impossible. Families reported the physical and psychological regression they observed in their loved ones due to the lack of stimulation usually provided during visits. For other patients, compensating for the prohibition on visits by using video calls, telephone calls, and text messages was greatly appreciated (for more details, see Additional file 4).

## Discussion

To the best of our knowledge, this research was the first to use a PREMs questionnaire to examine the impact of visiting restrictions on patients and their relatives in a hospital setting during the COVID-19 pandemic’s first wave in Switzerland. The Valais Hospital’s values, and those of its healthcare staff, recognize relatives’ important role in their loved one’s healthcare and hospital discharge trajectories. However, this was a very challenging period for patients, relatives, and staff, with unforeseen and unpredictable events, daily changes, and many restrictions. The sudden implementation of visiting restrictions destabilized the hospitalization process and relatives’ roles within that process. Obtaining an elevated response rate (75%) from the responding patients’ relatives was, therefore, not surprising as it offered them a chance to express both their positive and negative lived experiences of these extreme health circumstances.

This study was specifically conducted during the COVID-19 pandemic’s first wave, and considering relatives as essential partners in care—and not just as visitors—is part of the Valais Hospital staff’s mission. Relatives of a SARS-CoV-2-infected patient were more likely to have revealed how affected they were by the visiting restrictions than were relatives of non-infected patients. Relatives expressed their perceptions of ethical and clinical issues in their responses to the open-ended question. This was not surprising and was in line with Jaswaney et al*.*’s [50] findings that visiting restrictions can be problematic, creating many ethical issues related to who can and cannot visit. The impossibility of being physically present for their hospitalized loved ones created worry, anxiety, sadness, and a perceived greater need for more information and updates on the relative’s condition, as expressed in relatives’ comments and in line with the findings of Rottenburg et al. and Sahoo et al. [22, 23]. Many relatives reported stress due to uncertainty, and not being allowed into the hospital created emotional worries and feelings of failing to support and protect their kin. Being present at the patient’s bedside, on the other hand, helped relatives to understand and cope with situations, as reported in the recent study by Hochendoner et al. of the relatives of ICU patients [51]. Our findings revealed significant differences between the high impact of visiting restrictions perceived by the relatives of SARS-CoV-2-infected patients and the lower impact perceived by relatives of non-infected patients, and this effect was similar across ward types. As one might imagine, visiting restrictions strongly affected the relatives of patients in the gynecology/obstetrics, maternity, geriatrics, and general medicine wards, more so than in other hospitalization wards and in line with Hochendoner et al.’s study [51]. This was independent of patient age group or LOS and of relatives’ perceptions of the severity of a SARS-CoV-2 infection. Hoffman et al. used the example of oxygen supplementation to express how crucial contact is with healthcare staff who can explain the patient’s situation. The COVID-19 pandemic and the stresses involved were highly disturbing for relatives waiting for news on their loved ones. Although the majority of our participating relatives did feel considered by healthcare staff, not all of them did; some expressed concerns about visiting restrictions and felt less considered or not at all considered regarding involvement in the care provided. A more detailed analysis of each hospitalization would clarify those concerns, but that was beyond this paper’s scope. In opposition to some free comments criticizing a lack of information, our quantitative results showed that most relatives felt well informed by healthcare staff, with no difference between the relatives subjected to visiting restrictions and those not. However, some hospitalization wards showed significant differences, such as maternity/obstetrics, which was unsurprising and in line with recent publications by Venkatesh et al. and Hugelius et al. [52, 53]. Our linear regression model confirmed this, explaining the mild-to-moderate variance in the affect scores of relatives whose loved ones were hospitalized in general medicine and gynecology/obstetrics wards.

The Valais Hospital tried to replace physical visits with various digital and technical means, but these had clear limitations. Relatives subjected to visiting restrictions reported lower scores for the quality of communication than relatives who could visit. Unfortunately, relatives’ video or telephone meetings with patients in acute care settings led to fewer agreed changes to care goals with staff than did in-person meetings, as was confirmed in the recent studies by Reitzle et al., Lin et al., Sken et al., and Rose et al. [31–34]. Also, despite these substitute visiting methods, in-person visiting restrictions reduced relatives’ comprehension of the patient’s overall condition and their possibilities for maintaining social relations, as confirmed by Mahery et al. [27]. Based on relatives’ free comments, visiting restrictions were also a source of emotional distress and increased workloads for healthcare staff, who may not have agreed with hospital policies resulting in them spending a lot of time informing and communicating with relatives. This may have caused problems in the dialogue between healthcare staff and relatives and thus reduced the possibilities of ensuring consensus-based care [22].

The Valais Hospital regularly updated its visiting restrictions, referring closely to Swiss federal and cantonal public health policies concerning SARS-CoV-2 infection risk–benefit assessment—the cornerstone of medical and pandemic policy decision-making. It nevertheless remains difficult to determine whether those visiting restrictions were effective in limiting the spread of COVID-19. Although it might be reasonable to speculate that these policies slowed its spread, based on a mechanistic understanding of the disease, visiting restrictions should be weighed against the potential harm to patients. Our study highlighted the complexities associated with the numerous factors impacted by hospital visiting restrictions. Our results advocate for a more tailored, adaptable, and patient-centered approach to visiting restrictions depending on the clinical situation. Reasonable exceptions might include allowing fathers to visit labor and delivery rooms, pediatrics wards, and ICU units. The authors endorse a nuanced approach to hospital visiting restrictions, taking into account the patient population, visitors’ use of personal protective equipment, screening measures, community disease prevalence, and other circumstances. Visiting restrictions should be clearly and transparently communicated to relatives. Patient discharge during periods with visiting restrictions is another concern, as healthcare staff are tasked with establishing a critical partnership with relatives to organize discharge planning [54, 55].

The Valais Hospital and its staff worked to maintain strong relationships between patients and relatives, convinced that these improve the patient experience, safety, and outcomes. Visiting restrictions aimed to protect patients and staff, but some relatives felt that they were no longer essential partners in care. Most relatives understood the rapid shift to strict visiting restrictions, given the nature of the COVID-19 crisis. Nevertheless, these policies proved very difficult for relatives, causing significant emotional stress, concerns for patient safety, and the inability to support loved ones at the bedside. Relatives and healthcare staff must remain partners in care, even when challenging circumstances put that partnership under stress. The COVID-19 pandemic evolved rapidly and continues to do so. Many directives and shifts in policy were implemented without the opportunity to engage with relatives, including a shift in language that returned relatives to their roles as visitors rather than as partners in care. Effective and appropriate communication about policy changes and how relatives and healthcare staff can continue to work together as partners in care is essential to establishing trust and positive collaboration.

## Study strengths

To the best of our knowledge, this was the first PREMs carried out in Switzerland to include hospitalized patients’ relatives within the context of the COVID-19 pandemic. The study employed as many psychometrically validated questions as possible to investigate PREMs appropriately among relatives.

## Study limitations

The study also had some limitations. A first limitation is the inability to interpret it outside the context of the COVID-19 pandemic’s first wave. Valais Hospital had never conducted a PREMs survey and, to the best of our knowledge, no similar studies of relatives’ experiences were conducted during this period, making comparisons with our results difficult. The survey’s self-reporting questionnaire was designed especially for the present study; however, the internal consistency of the PREMS questions on visiting restrictions was limited, and no comparison with the original calculation was available.

Other significant limitations to our survey were the reliability and validity of the PREMs self-reported questionnaire employed. The internal consistency of the five unidimensional questions used in it—(i) Loved ones’ levels of affection due to visiting restrictions? (ii) How serious do you think the COVID-19 pandemic is? (iii) How did you perceive the information you received about the COVID-19 pandemic during your loved one’s hospital stay? (iv) How would you rate communication with the staff? (v) As a close relative, how did the hospital staff treat you?—was not tested. At that time, a trade-off between urgency and the scientific accuracy of using a self-reporting questionnaire did not allow us the time to test the PREMs questionnaire’s reliability, especially these unidimensional questions. Moreover, the questionnaire’s limited validity could not be assessed or attenuated by correlating its scores and results with a similar instrument as this did not exist when the survey was launched during the COVID-19 pandemic’s first wave.

Another limitation was the delay of 4 to 6 months between patients’ hospitalization and their self-reported survey responses. Furthermore, since well before the COVID-19 pandemic, the Valais Hospital had systematically invited patients to share their opinions and rate their satisfaction with the hospital’s organization and performance; the present survey did not investigate relatives’ satisfaction so as to avoid redundancies, and this could be considered a limitation. Studies based on PREMs are usually regarded as a low level of evidence, as survey completion may lack rigor and the accuracy of the information provided cannot be verified. In addition, the content of the concepts explored has still not been standardized, and we could have missed some relevant experiences among relatives.

To respect healthcare’s Quadruple Aim, further research among healthcare professionals should complement this study. Based on our results and in line with the existing international literature published after the COVID-19 pandemic’s first-wave visiting restrictions, restricting visits by all the relatives of hospitalized patients is not recommendable [3, 56]. Future policies must clearly incorporate patients’ and relatives’ insights on this topic. Detailed evaluations of restrictions based on hospital settings (e.g., emergency departments, maternity, psychiatry, and surgery wards) are needed to quantify the relevant risks of visitor absence.

## Conclusion

The present study described relatives’ experiences of visiting restrictions, how they were affected by these, and their perceptions of the severity of a SARS-CoV-2 infection and of information flow and communication during the COVID-19 pandemic’s first wave. About two-thirds of responding relatives were moderately emotionally affected by the visiting restrictions, and most felt well-considered by the healthcare staff.

Responses to our survey’s open question showed the unique aspects of each relative’s experiences of their loved one’s hospitalization. Our patient-reported experience measures survey (PREMs) data revealed COVID-19’s impact on the social determinants of health among patients’ relatives, thus helping to identify opportunities for improving patient-centered care throughout the following waves of this ongoing crisis and perhaps after it.

Although the PREMs questionnaire collected interesting data on relatives’ experiences of visiting restrictions during the COVID-19 pandemic’s first wave, our results should be interpreted with caution considering the regional nature of the health conditions examined and the limitations in the consistency of our ad hoc questionnaire. Future research will need to focus on embedding the collection of PREMs more broadly throughout healthcare institutions, increasing the use of their findings by patients, relatives, clinicians, and policymakers, and facilitating comparisons of patient-reported experiences internationally.

### Supplementary Information

**Additional file 1.**

**Additional file 2.** Relatives’ perceptions about the information received on their hospitalized loved one.

**Additional file 3.** Relatives’ evaluation of the quality of communication.

**Additional file 4.** Content analysis of relatives’ comments about visiting restrictions as applied across different hospitalization units and departments (*n* = 71).

## Data Availability

The dataset used and analyzed during the current study is available from the corresponding author upon reasonable request.
