# Supplementary material for: Relatives’ experiences of visiting restrictions during the COVID-19 pandemic’s first wave: a PREMs study in Valais Hospital, Switzerland
Source: BMC Health Serv Res. 2023 Sep 19;23:1008. doi: 10.1186/s12913-023-10013-9 (PMC10510254; doi:10.1186/s12913-023-10013-9)
Supplement: Supplementary file 1 — Additional file 1. [file 12913_2023_10013_MOESM1_ESM.pdf]

Survey of your lived experiences as a patient and of those of your family caregivers throughout your hospitalisation during the COVID-19 pandemic. (from 27 February to 11 May)

PART 1

1. **Personal information** (please complete and tick)
    - Year of birth: …………...
    - Sex: □ Female □ Male □ Other
    - Marital status: □ Married □ Single □ Divorced/Separated
    - Do you live alone? □ Yes □ No
    - What is your highest level of education?
      □ Compulsory schooling □ High school (and/or an apprenticeship)
      □ Higher studies
    - Was a family member (and/or family caregiver) involved throughout your hospitalisation?
      □ Yes □ No
      If yes, and you agree, the questions on pages 7 and 8 should be filled in by your family caregiver and then returned in the same envelope as your answers.
2. **Date of hospital admission** (day/month): …………..
3. **Date of hospital discharge** (day/month): ……..…….
4. **Please tick the different hospital departments that you attended during your hospitalisation**:
    - □ An advanced medical post (e.g. a tent outside the hospital)
    - □ Emergency department
    - □ Medical care department
    - □ Surgery department
    - □ Psychiatry department
    - □ Gynaecology/maternity department
    - □ Continuous care unit
    - □ Intensive care unit
    - □ Rehabilitation unit
    - □ I don't know

5. **Were you hospitalised because of an infection by COVID-19?**
   □ Yes □ No □ I don't know

6. **How did you perceive the information you received about the COVID-19 pandemic during your hospital stay?** (please tick one box only)
    - □ Totally inadequate
    - □ Inadequate
    - □ Slightly inadequate

- ◻ No opinion (neutral)
- ◻ Just adequate enough
- ◻ Adequate
- ◻ Very adequate

7. **How would you rate the hospital's communication?** (please tick one box only)

Poor	Passable	Good	Very good	Excellent
◻	◻	◻	◻	◻

8. **With regards to the information that you needed throughout your hospitalisation, which of the subjects below were <u>the most important</u> to you?** (please tick a maximum of 3 boxes)
   - ◻ The means of COVID-19 transmission
   - ◻ The incubation time of COVID-19 (the time between infection and the appearance of the first symptoms)
   - ◻ The symptoms of COVID-19
   - ◻ The preventive measures to avoid contracting COVID-19
   - ◻ The risks of contracting COVID-19
   - ◻ The risks of falling severely ill
   - ◻ The treatments for COVID-19
   - ◻ I did not need any other information
   - ◻ Other: ……………………………………………………

9. **Did you feel safe throughout your hospital stay?** (please tick a number between 1 and 10 to give your opinion on your feelings of safety, with 10 being that you felt totally safe and 1 being that you felt totally unsafe)

Totally unsafe Totally safe

1	2	3	4	5	6	7	8	9	10

10. **How serious do you think the COVID-19 pandemic is?**

Not at all serious	Not very serious	Slightly serious	Serious	Very serious
◻	◻	◻	◻	◻

11. **The following questions are about the trust you felt in your physicians and nurses throughout your hospital stay during the <u>pandemic situation</u>.**
Tick the box which best corresponds to your answer to each question.

    a) I doubt that the health-care staff really cares about me as a person.
    ☐ Totally disagree ☐ Disagree ☐ No opinion ☐ Agree ☐ Totally agree

    b) The health-care staff is usually considerate of my needs and makes them a priority.
    ☐ Totally disagree ☐ Disagree ☐ No opinion ☐ Agree ☐ Totally agree

c) I have a lot of trust in health-care staff, and I always try to follow their advice.

☐ Totally disagree ☐ Disagree ☐ No opinion ☐ Agree ☐ Totally agree

d) If health-care staff tell me that this is the way it is, then it must be true.

☐ Totally disagree ☐ Disagree ☐ No opinion ☐ Agree ☐ Totally agree

e) I sometimes distrust health-care staff's opinions and like to get a second opinion.
☐ Totally disagree ☐ Disagree ☐ No opinion ☐ Agree ☐ Totally agree

f) I trust health-care staff's judgement on my medical care.
☐ Totally disagree ☐ Disagree ☐ No opinion ☐ Agree ☐ Totally agree

g) I feel that health-care staff do not do everything they should concerning my medical treatments.
☐ Totally disagree ☐ Disagree ☐ No opinion ☐ Agree ☐ Totally agree

h) Health-care staff are real experts in the management of medical problems like mine.
☐ Totally disagree ☐ Disagree ☐ No opinion ☐ Agree ☐ Totally agree

i) I trust that health-care staff will tell me if a mistake has been made in my treatment.
☐ Totally disagree ☐ Disagree ☐ No opinion ☐ Agree ☐ Totally agree

j) I am sometimes afraid that health-care staff will not keep the information we discuss confidential.
☐ Totally disagree ☐ Disagree ☐ No opinion ☐ Agree ☐ Totally agree

12. **Were you aware that psychological support was available to you throughout your hospital stay?**
□ Yes □ No

13. **Did you receive any psychological support throughout your hospitalisation?**
□ Yes □ No □ I don't know

If you did benefit from psychological support, were you satisfied with that service?

Not at all satisfied	Not very satisfied	Satisfied	Very satisfied	Extremely satisfied

◻	◻	◻	◻	◻

Do you think that you still need psychological support today?

◻ Yes ◻ No ◻ I don't know

14. **Did your health problem, combined with your hospitalisation during a period when the canton of Valais was facing a pandemic, cause you to feel stressed?**
Please respond to the following 10 questions as spontaneously as possible.

**In the last month, how often:**	**Never**	**Almost never**	**Sometimes**	**Fairly often**	**Very often**
1. Have you been upset because of something that happened unexpectedly?	◻	◻	◻	◻	◻
2. Have you felt that you were unable to control the important things in your life?	◻	◻	◻	◻	◻
3. Have you felt nervous or stressed?	◻	◻	◻	◻	◻
4. Have you felt confident in being able to face up to your problems?	◻	◻	◻	◻	◻
5. Have you felt that things were going the way you wanted them to?	◻	◻	◻	◻	◻
6. Have you felt overwhelmed by all of the things that you had to get done?	◻	◻	◻	◻	◻
7. Have you felt capable of managing the inconveniences you encounter?	◻	◻	◻	◻	◻
8. Have you felt that you were functioning to the best of your capacities?	◻	◻	◻	◻	◻
9. Have you felt annoyed because events were outside of your control?	◻	◻	◻	◻	◻
10. Have you felt that your difficulties were so enormous that you could not control them?	◻	◻	◻	◻	◻

15. **Was there anything missing or lacking from your hospital discharge?**

□ No □ Yes If yes, please describe what:

………

16. **Do you still have any physical or other symptoms linked to an infection with COVID-19?**

□ No □ Yes If yes, please describe which ones:

………

………

17. **Do you have any improvements to suggest?**

……

……

……

We sincerely thank you for your participation.

Please return this questionnaire in the attached stamped, addressed envelope.

PART 2

This part of the questionnaire should be filled in by the person who acted as your **close family caregiver** throughout your hospitalisation. If you did not have family members directly involved throughout your hospitalisation, please leave this section blank.

18. **Were you able to visit your relation in hospital?**
    - □ Yes
    - □ No

If not, how did you manage to maintain contact with your relation? (you may tick more than one box)

- □ Telephone with the patient
- □ Telephone with professional caregivers
- □ Email
- □ Other: …………………………………………………

If not, did this affect you? (please tick one answer only)

- □ I was not affected
- □ I was slightly affected
- □ No opinion
- □ I was moderately affected
- □ I was very affected

19. **How did you perceive the information you received about the COVID-19 pandemic throughout your relation's hospital stay?** (please tick one box only
    - □ Totally inadequate
    - □ Inadequate
    - □ Slightly inadequate
    - □ No opinion (neutral)
    - □ Just adequate enough
    - □ Adequate
    - □ Very adequate

20. **How would you rate communication with the staff?** (please tick one box only)
    - □ Poor
    - □ Passable
    - □ Good
    - □ Very good
    - □ Excellent

21. **With regards to the information needed throughout your relation's hospitalisation, which of the subjects below were <u>the most important</u> to you?** (please tick a maximum of 3 boxes)
    - ☐ The means of COVID-19 transmission
    - ☐ The incubation time of COVID-19 (the time between infection and the appearance of the first symptoms)
    - ☐ The symptoms of COVID-19
    - ☐ The preventive measures to avoid contracting COVID-19
    - ☐ The risks of contracting COVID-19
    - ☐ The risks of falling severely ill
    - ☐ The treatments for COVID-19
    - ☐ I did not need any other information
    - ☐ Other: …………………………………………………

22. **As a close family caregiver, how did the hospital staff consider you?**
    (please tick one box only)
    - ☐ I was not considered at all
    - ☐ I was moderately considered
    - ☐ I was fully considered

23. **How serious do you think the COVID-19 pandemic is?**

Not at all serious	Not very serious	Slightly serious	Serious	Very serious
☐	☐	☐	☐	☐

Would you like to add any comments about your experience of your relation's hospitalisation during the pandemic?

....

....

....

We sincerely thank you for your participation.
