# Supplementary material for: Relatives’ experiences of visiting restrictions during the COVID-19 pandemic’s first wave: a PREMs study in Valais Hospital, Switzerland
Source: BMC Health Serv Res. 2023 Sep 19;23:1008. doi: 10.1186/s12913-023-10013-9 (PMC10510254; doi:10.1186/s12913-023-10013-9)
Supplement: Supplementary file 2 — Additional file 2. Relatives’ perceptions about the information received on their hospitalized loved one. [file 12913_2023_10013_MOESM2_ESM.docx]

**Table Additional file 2.** Relatives’ perceptions about the information received on their hospitalized loved one.

| **Variables** | **Median (IQR 1–3)** | ***P*-value** |
| --- | --- | --- |
| **Relatives** |  |  |
| Visit restrictions (n = 510) | 5 (6) |  |
| No visit restrictions (n = 265) | 6 (6) | 0.001 |
| **SARS-CoV-2** |  |  |
| **Visiting restrictions** |  |  |
| SARS-CoV-2-infected patients (n = 95) | 6 (6) | 0.254 |
| Non-infected patients (n = 07)) | 5 (6) |  |
| **No visiting restrictions** |  |  |
| SARS-CoV-2-infected patients (n = 10) | 6 (7) | 0.929 |
| Non-infected patients (n = 252) | 6 (6) |  |
| **Age groups (years)** |  |  |
| **Visiting restrictions** |  |  |
| 18–34 (n = 66) | 4 (6) | 0.248 |
| 35–55 (n = 91) | 5 (6) |  |
| 56–64 (n = 72) | 4 (6) |  |
| 65–74 (n = 104) | 6 (6) |  |
| 75 or more (n = 138) | 6 (6) |  |
| **No visiting restrictions** |  |  |
| 18–34 (n = 59) | 5 (6) | 0.388 |
| 35–55 (n = 55) | 6 (6) |  |
| 56–64 (n = 29) | 6 (7) |  |
| 65–74 (n = 46) | 5 (7) |  |
| 75 or more (n = 58) | 6 (6) |  |
| **Hospitalization ward** |  |  |
| **Visiting restrictions** |  |  |
| Surgery (n = 89) | 6 (6) | < 0.001 |
| General Medicine (n = 112) | 5.5 (6) |  |
| Gynecology/obstetrics (n = 58) | 4 (6) |  |
| Intermediate care/ICU (n = 107) | 6 (6) |  |
| Unknown trajectory (n = 91) | 5 (6) |  |
| Psychiatry (n = 11) | 4 (6) |  |
| Rehabilitation/geriatrics (n = 9) | 2 (6) |  |
| Multiple wards other than ICU (n = 33) | 6 (6) |  |
| **No visiting restrictions** |  |  |
| Surgery (n = 50) | 6 (6) | 0.030 |
| General Medicine (n = 39) | 6 (7) |  |
| Gynecology/obstetrics (n = 66) | 6 (6) |  |
| Intermediate care/ICU (n = 46) | 6 (6) |  |
| Unknown trajectory (n = 30) | 6 (7) |  |
| Psychiatry (n = 13) | 3 (6) |  |
| Rehabilitation/geriatrics (n = 5) | 2 (6) |  |
| Multiple wards other than ICU (n = 16) | 6 (6) |  |
| **Length of stay (days)** |  |  |
| **Visiting restrictions** |  |  |
| 1–14 (n = 322) | 6 (6) | 0.220 |
| ≥ 15 (n = 110) | 6 (6) |  |
| **No visiting restrictions** |  |  |
| 1–14 (n = 159) | 6 (6) | 0.564 |
| ≥ 15 (n = 50) | 6 (6) |  |

1 = No opinion, 2 = Completely inappropriate, 3 = Inappropriate, 4 = Slightly inappropriate,

5 = Mildly appropriate, 6 = Moderately appropriate, 7 = Completely appropriate
