# Supplementary material for: Relatives’ experiences of visiting restrictions during the COVID-19 pandemic’s first wave: a PREMs study in Valais Hospital, Switzerland
Source: BMC Health Serv Res. 2023 Sep 19;23:1008. doi: 10.1186/s12913-023-10013-9 (PMC10510254; doi:10.1186/s12913-023-10013-9)
Supplement: Supplementary file 3 — Additional file 3. Relatives’ evaluation of the quality of communication. [file 12913_2023_10013_MOESM3_ESM.pdf]

Additional File 3

Table Additional file 3. Relatives' evaluation of the quality of communication

Variables	Poor	Acceptable	Good	Very good	Excellent	Median (IQR 1–3)	*P*-value
**Relatives (n = 790)**	86 (10.9)	87 (11)	288 (36.5)	228 (28.9)	101 (12.8)	3 (4)	
Visiting restrictions (n = 501)	67 (13.4)	64 (12.8)	195 (38.9)	125 (25)	50 (10)	3 (4)	< 0.001
No visiting restrictions (n = 289)	19 (6.6)	23 (8)	93 (32.2)	103 (28.9)	51 (17.6)	4 (4)	
**Visiting restrictions**							
Infected COVID patients (n = 93)	14 (15.1)	11 (11.8)	26 (28)	32 (34.4)	10 (10.8)	3 (4)	0.094
Non-infected COVID patients (n = 399)	52 (13)	52 (13)	165 (41.4)	91 (22.8)	39 (9.8)	3(4)	
**No visiting restrictions**							
Infected COVID patients (n = 11)	0	0	2 (18.2)	6 (54.5)	3 (27.3)	4 (5)	0.404
Non-infected COVID patients (n = 274)	19 (6.9)	22 (8)	89 (32.5)	97 (35.4)	47 (17.2)	4 (4)	
**Age group (years)**							
**Visiting restrictions**							
18–34 (n = 62)	12 (19.4)	14 (22.6)	21 (33.9)	12 (19.4)	3 (4.8)	3 (3)	0.147
35–55 (n = 84)	15 (17.9)	13 (15.5)	35 (41.7)	14 (16.7)	7 (8.3)	3 (3)	
56–64 (n = 69)	11 (15.9)	7 (10.1)	28 (40.6)	16 (23.2)	7 (10.1)	3 (4)	
65–74 (n = 102)	13 (12.7)	11 (10.8)	36 (35.3)	33 (32.4)	9 (8.8)	3 (4)	
75 or more (n = 142)	12 (8.5)	16 (11.3)	56 (39.4)	39 (27.5)	19 (13.4)	3 (4)	
**No visiting restrictions**							
18–34 (n = 60)	7 (11.7)	8 (13.3)	16 (26.7)	19 (31.7)	10 (16.7)	3 (4)	0.709
35–55 (n = 58)	5 (8.6)	3 (5.2)	21 (36.2)	22 (37.9)	7 (12.1)	3 (4)	
56–64 (n = 29)	1 (3.4)	2 (6.9)	8 (27.6)	14 (48.3)	4 (13.8)	4 (4)	
65–74 (n = 52)	1 (1.9)	5 (9.6)	17 (32.7)	19 836.5)	10 (19.2)	4 (4)	
75 or more (n = 69)	3 (4.3)	5 (7.2)	23 (33.3)	24 (34.8)	14 (20.3)	4 (4)	
**Hospitalization ward**							
**Visiting restrictions**							
Surgery (n = 82)	8 (9.8)	16 (19.5)	32 (39)	15 (18.3)	11 (13.4)	3 (4)	0.045
General Medicine (n = 110)	17 (15.5)	10 (9.1)	48 (43.6)	26 (23.6)	9 (8.2)	3 (4)	
Gynecology/obstetrics (n = 59)	15 (25.4)	9 (15.3)	23 (39)	10 (16.9)	2 (3.4)	3 (3)	
Intermediate care/ICU (n = 109)	11 (10.1)	17 (15.6)	36 (33)	32 (29.4)	13 (11.9)	3 (4)	
Unknown trajectory (n = 90)	10 (11.1)	6 (6.7)	34 (37.8)	29 (32.2)	11 (12.2)	3 (4)	
Psychiatry (n = 34)	3 (27.3)	1 (9.1)	5 (45.5)	1 (9.1)	1 (9.1)	3 (3)	
Rehabilitation/geriatrics (n = 9)	1 (11.1)	3 (33.3)	4 (44.4)	0	1 (11.1)	3 (3)	
Multiple wards other than ICU (n = 31)	2 (6.5)	2 (6.5)	13 (41.9)	12 (38.7)	2 (6.5)	3 (4)	
**No visiting restrictions**							
Surgery (n = 55)	6 (10.9)	4 (7.3)	21 (38.2)	19(34.5)	5 (9.1)	3 (4)	0.701
General Medicine (n = 46)	2 (4.3)	3 (6.5)	12 (26.1)	19 (41.3)	10 (21.7)	4 (4)	
Gynecology/obstetrics (n = 67)	4 (6)	6 (9)	19 (28.4)	28 (41.8)	10 (14.9)	4 (4)	
Intermediate care/ICU (n = 48)	3 (6.3)	3 (6.3)	17 (35.4)	18 (37.5)	7 (14.6)	4 (4)	
Unknown trajectory (n = 36	2 (5.6)	2 (5.6)	15 (41.7)	8 (22.2)	9 (25)	3 (4)	
Psychiatry (n = 14)	1 (7.1)	3 (21.4)	4 (28.6)	4 (28.6)	2 (10)	3 (4)	
Rehabilitation/geriatrics (n = 6)	0	1 (16.7)	1 (16.7)	1 (16.7)	3 (50)	4 (5)	
Multiple wards other than ICU (n = 17)	1 (5.9)	1 (5.9)	4 (23.5)	6 (35.3)	5 (29.4)	4 (5)	
**Length of stay (days)**							
**Visiting restrictions**							
1–14 (n = 310)	10 (9)	14 (12.6)	40 (36)	36 (32.4)	11 (9.9	3 (4)	0.306
≥ 15 (n = 111	48 (15.5)	39 (12.6)	121 (39)	76 (24.5)	26 (8.4)	3 (4)	
**No visiting restrictions**							
1–14 (n = 169)	13 (7.7)	13 (7.7)	56 (33.1)	64 (37.9)	23 (13.6)	4 (4)	0.199
≥ 15 (n = 56)	2 (3.6)	4 (7.1)	15 (26.8)	20 (35.7)	15 (26.8)	4 (5)	
