# Supplementary material for: Relatives’ experiences of visiting restrictions during the COVID-19 pandemic’s first wave: a PREMs study in Valais Hospital, Switzerland
Source: BMC Health Serv Res. 2023 Sep 19;23:1008. doi: 10.1186/s12913-023-10013-9 (PMC10510254; doi:10.1186/s12913-023-10013-9)
Supplement: Supplementary file 4 — Additional file 4. Content analysis of relatives’ comments about visiting restrictions as applied across different hospitalization units and departments (n = 71). [file 12913_2023_10013_MOESM4_ESM.pdf]

Additional file 4.

Content analysis of relatives' comments about visiting restrictions as applied across different hospitalization units and departments (n = 71).

Unit/ Department	N°*	Lived experience	Comments about the situation
Maternity	13	In the case of childbirth, the father's place–who could have been tested before–is next to the mother and the child. Don't you think?	The visiting restrictions mainly affected the maternity and pediatrics department, with fathers being forbidden to participate in deliveries and to visit their wives and newborns. Fathers were initially excluded from the preparations for labor, which caused a lot of frustration and stress for both mothers and fathers. Although they understood the preventive measures against the coronavirus, maternity patients and their relatives, who were not classified as sick patients, found these restrictions too extreme. The issue of testing to be able to participate in the entire delivery process was mentioned in the comments. Limitations to or even prohibitions on visits by fathers were not well received, especially time limitations. Being deprived of this life experience and unable to provide support to the mother or to see the child at its birth and during its first days of life was a very bad experience, leaving fathers with intense regrets.
Neonatology	1	Understanding the hospital sector's state of stress of the hospital world...I had expected a different appreciation of priorities...For me, a hospitalization in neonatology should ensure the right to visits no matter what...	The limitations to or even prohibitions on visits to the neonatology unit was very badly received by relatives, and they did not understand that the measures to limit contamination by SARS-CoV-2 concerned every department in the Valais Hospital.
Prohibition on accompanying patients during emergency and non-emergency admissions	7	It is tough to leave a loved one–especially my sick wife–outside the door without accompanying her or supporting her during these difficult moments, but I understand the measures taken.	The prohibition on visits also included a prohibition on accompanying loved ones during hospital admissions. This moment of separation, leaving loved ones to the unknown, aroused the very strong emotions of worry, anxiety, stress, fears of not seeing them again, and apprehension while waiting for news. Not being able to support the suffering patient in hospital was perceived as very difficult. Relatives would have liked to have had a waiting area on site.
Impact of visiting restrictions on frail or vulnerable patients	30	The ban on visits is traumatic for all relatives.	Visiting restrictions were very badly received by relatives and frail patients, especially regarding patients with Alzheimer's, cognitive disorders, or at the end of life, with whom video calls were complicated or impossible. Families noted the feelings of abandonment or loneliness expressed to them by patients. Families also reported their loved ones' physical and psychological regression due to the lack of stimulation that usually occurred during visits. For other patients, compensating for the prohibitions on visiting with video calls, telephone calls, and text messages was greatly appreciated.
Lack of contact with hospital staff	18	Very difficult because there is no information, no visits, and the husband is alone at home. Very complicated.	The hospital staff gave some families little or no information about their loved one's condition. This was even more difficult to manage because families were kept at a distance by prohibitions or restrictions on visiting.

		Lack of information for the person at home.	
Prohibition on contacting staff	2	We were told not to call more than once a day because they were too busy. The situation was very complicated at the hospital, but it's hard to have so little news when both parents are sick and almost unreachable.	Some family members noted that hospital staff told them not to call to check on patients. Given the exceptional situation caused by the pandemic and the stress involved, this was disturbing for relatives waiting to hear news of their loved ones.
